# Characterization of physiological and molecular processes associated with potato response to Zebra chip disease

**DOI:** 10.1038/hortres.2017.69

**Published:** 2017-12-06

**Authors:** Chika C Nwugo, Venkatesan G Sengoda, Li Tian, Hong Lin

**Affiliations:** 1 San Joaquin Valley Agricultural Sciences Center, USDA-ARS, Parlier, CA 93658, USA; 2 California Seed and Plant Labs, Sacramento, CA 95668, USA; 3 USDA-ARS, Yakima Agricultural Research Laboratory, Wapato, WA 98951, USA; 4 Department of Plant Sciences, University of California, Davis, Davis, CA 95616, USA

## Abstract

Transcriptional analyses identified molecular mechanisms associated with the response of leaf and root potato tissues to ‘*Candidatus.* Liberibacter solanacearum’ (Lso) infection, presumptive causal agent of zebra chip disease (ZC). Putative Lso infection affected several host processes including defense response-, regulation-, starch metabolism- and energy production-related processes. Interestingly, while proteinase inhibitors were strongly upregulated in leaf tissues, a concomitant downregulation was observed in root tissues. Quantitative polymerase chain reaction (qPCR) analysis suggests that alternative splicing might play a role. Furthermore, the transcriptional expression of redox homeostasis-related genes, including superoxide dismutase, showed the most inconsistent response to Lso in leaf and root tissues, highlighting potential targets of Lso susceptibility. Additionally, a net increase in gene expression in ZC-affected tissues despite the concomitant downregulation of photosynthesis-related processes, suggests a putative Lso-mediated low resource-use-efficiency. Subsequent nutritional analyses revealed a hypothesized Lso-mediated increase in nutrient accumulation, particularly a 210 and 108% increases in the potassium concentration of ZC-affected leaf and root tissues, respectively, suggesting an important role for potassium in ZC pathophysiology. This study highlights insights of above and below ground tissues in molecular and physiological aspects associated with potato response to ZC.

## Introduction

Potato (*Solanum tuberosum* L.) is one of the most economically important non-grain crops. Zebra chip (ZC) is an emerging disease that affects all cultivated varieties of potato, resulting in significant revenue losses to commercial potato growers in the United States, Mexico, Central America and New Zealand.^1–3^ ZC is associated with the psyllid *Bactericera cockerelli* (Šulc), which harbors ‘*Candidatus* Liberibacter solanacearum’ (Lso), a presumptive gram-negative phloem-limited α-proteobacterium.^1,4–7^ Although Koch’s postulates have not been fulfilled due to the non-culturable attribute of Lso, there is a consensus agreement that Lso is etiologically associated with ZC.^1,6^ Accordingly, Lso-infected potato plants routinely show ZC symptoms, such as leaf curling, leaf chlorosis, leaf scorching, starch accumulation in vines and dark striping of fried tuber slices.^8–10^

Presently, the only effective ZC management strategy is the application of insecticides targeted against the insect vector. However, this method is neither economically nor environmentally sustainable because psyllid-infested fields require spray treatments at an increasing frequency per season, suggesting that a development of insecticide resistance in *B. cockerelli* is imminent due to the high fecundity and short generation time of the psyllid.^3^ While all commercially cultivated potato varieties are susceptible to ZC,^3^ understanding the host molecular response patterns associated with the disease could facilitate the identification of key ZC-affected potato interactions that may be applied towards disease management strategies for breeding or genetic engineering purposes.

ZC is a relatively new disease,^7,11^ but is etiologically and symptomatically similar to the highly destructive citrus huanglongbing (HLB) disease.^12^ Similar to ZC, HLB is associated with a non-culturable, psyllid-transmissible Liberibacter, ‘*Candidatus.* Liberibacter asiaticus’ (Las), and like ZC, HLB-affected stems show abnormally high levels of starch accumulation.^10,13^ Potatoes are annual plants and visibly respond faster to Lso infection compared to citrus response to Las infection.^14^ Thus, potato plants are potential viable, efficient and practical models for understanding the mechanisms involved in host response to Liberibacter-associated infections.

Previous studies by Wallis *et al.*^11^ showed that ZC-affected potatoes caused an increase in the levels of phenolics and amino acids, in addition to an increase in the activities of chitinase and polyphenol oxidase in ZC-affected potato tubers compared to healthy tubers. A subsequent study by Alvarado *et al.*^10^ showed a putative Lso-mediated increase in total protein production, the accumulation of starch and phenolics as well as an increase in polyphenol oxidase activity in potato vines and suggested that ZC disease development involves the reprogramming of potato stems to exhibit tuber-like properties. However, there is a paucity of information in the literature on the global gene regulatory processes associated with leaf and root tissue response of potato plants to putative Lso infection. Such information would be invaluable in elucidating the molecular basis and/or extent of the hypothesized Lso-mediated reprogramming of potato tissues and would shed light on the localized as well as ubiquitous (whole-plant) molecular processes associated with potato response to putative Lso infection.

Additionally, studies have highlighted a close relationship between nutrient concentrations concurrent to degrees of the severity of disease manifestation in plants, and altered nutritional balance in plants have become a fundamental aspect of the physiological responses of plants to diseases.^15^ For example, while investigating the yellowing disease of Norway spruce, Nechwatal and Osswald^16^ showed that microbes are associated with severe root damage which contributes to Mg^2+^ and Ca^2+^ deficiency in yellowing spruce compared to healthy spruce. Furthermore, we recently showed that K^+^ accumulation is associated with citrus response to HLB infection ^17,18^ and considering the earlier mentioned similarities between HLB and ZC, it would be interesting to investigate if a similar response exists in ZC-affected plants.

In this study, comparative high-throughput mRNA sequencing (RNA-Seq) analyses coupled with quantitative PCR (qPCR) analyses were performed to generate the global transcriptional profiles of ZC-affected leaf and root potato tissues. Meanwhile, ICP spectroscopy was used to investigate the nutritional effects of putative Lso-affection in ZC-affected leaf and root potato tissues. Results revealed novel insights into tissue-specific and whole-plant transcriptional gene regulatory mechanisms associated with ZC pathophysiology in addition to potential interrelationships between gene expression and nutrient concentration, which together, shed light on the molecular plant-microbial interactions involved in Liberibacter-associated plant diseases.

## Materials and methods

### Plant growth and infection

Disease-free potato mini-tubers of variety Atlantic (CSS Farms Inc., Colorado City, CO) were grown in ½-liter pots (Kord Products, Toronto, Ontario, Canada) containing a mixture of 86% sand, 13.4% peat moss, 0.5% Apex time-release fertilizer (J. R. Simplot Co., Lathrop, CA, USA), and 0.1% Micromax micronutrients (Scotts Co., Marysville, OH). The greenhouse was maintained at 24–28 °C, 50% RH, and 16:8 (Light:Dark) h photoperiod. Ten potato plants for each treatment group were grown on greenhouse benches in a completely randomization design. Four plants from each group were selected for RNA-Seq based on symptomatic expression and bacterial titers measured by qPCR. These four plants in each group represented the best biological replicates. Potato psyllid, *Bactericera cockerelli* (Šulc) colonies originally collected from a potato field in Dalhart, TX, USA, late fall in 2007, were reared on potato plants for several generations in a controlled environment: 29 °C, 50% RH, and 16:8 (Light:Dark) h photoperiod. Insects in the colonies were confirmed to be Lso positive monthly via PCR and 80 to 100% of psyllids were Lso-positive. To minimize the effect of psyllid feeding, potato plants (3–4 weeks old) were inoculated with putative Lso by exposure to Lso-positive adult potato psyllids (10 psyllids/plant) for 48 h. Insects were eliminated by treating plants with methyl bromide for 2 h in fumigation chamber. The presence of putative Lso in plants was determined by PCR. Three weeks after inoculation, plant tissues were collected from each plant and grouped into leaf tissues and root tissues comprising of small tubers. Samples were immediately frozen in liquid nitrogen, ground to a powder (6850 Freezer/Mill, Wolf Laboratories Ltd., UK) and stored in 80 °C until further analysis. The plant growth and inoculation experiments were performed at the USDA-ARS at Yakima Agricultural Research Laboratory, Wapato, WA, USA.

### Comparative transcriptomics analyses

Global transcriptional expression analysis was done in two major steps encompassing RNA-Seq and qPCR analyses. For RNA-Seq analyses, total RNA was extracted from leaf and root tissues of four replicate healthy or ZC-affected potato plants using TRIZol reagent according to the manufacturer’s protocol (Invitrogen, Life Technologies, Grand Island, NY, USA). The quality and quantity of the isolated total RNA was verified using an Agilent 2100 bioanalyzer and was quantified by Quant-iT RiboGreen RNA Assay Kit (Invitrogen, Life Technologies). Extracted total RNA samples from each replicate leaf and root tissues in ZC-affected and healthy groups were subjected to RNA-Seq analyses, which was performed at the UC Davis Genome Centre Expression Analysis Core using either Illumina’s Genome Analyzer II Sequencing System or the HiSeq 2000 (Illumina San Diego, CA, USA). RNA-Seq libraries were generated for healthy and ZC-affected leaf and root tissues and mapped to potato transcript sequences. The number of reads/transcripts for each coding region was determined, normalized against total reads between libraries generated from healthy or ZC-affected tissues and the ratio of reads between healthy and Lso-infected tissues was calculated. Genes that showed at least a two-fold change at 95% confidence interval were considered to be differentially expressed and subjected to further validation by qPCR.

For qPCR analyses, total RNA extraction was performed and repeated three times per sample from same plants used for RNA-Seq analyses. cDNAs were generated using the SuperScript First-Strand Synthesis System for RT-PCR (Invitrogen, Life Technologies, USA). Gene specific primers were designed using Primer Express (Applied Biosystems, Foster City, CA, USA) and the list of primers used in this study is provided in Supplementary Table S1. Samples were prepared according to manufacturer’s protocol. qPCR was performed using SYBR-Green PCR Master Mix (Applied Biosystems, CA) on a ViiA™ 7 Real-Time PCR System (Applied Biosystems, CA, USA) with the following cycle conditions: 95 °C for 10 min followed by 40 cycles of 95 °C for 15 s, 55 °C for 45 s and 72 °C for 45 s, which was accompanied by dissociation analysis settings as follows: 95 °C for 15 s, 60 °C for 15 s and 95 °C for 15 s. Dissociation curves were carefully examined to confirm the absence of signals from non-specific PCR products. Relative number of transcripts per target gene was obtained using the ΔΔ*C*_t_ method.^19^ An actin gene (gi|1498371) was used as the endogenous control/normalizer after multiple candidates were tested based on suggestions by Nicot *et al.*^20^ Gene expression data were further analyzed. Where necessary, qPCR amplified gene products were gel-purified on 1% agarose, extracted (QIAquick PCR purification kit, Qiagen, USA) and re-sequenced (BigDye Terminator v3.1 sequencing kits, Life Technologies, USA) by an ABI 3130 Genetic Analyzer (Applied Biosystems).

### Nutrient content analysis

The concentrations of the following cationic macronutrients: calcium (Ca), potassium (K), magnesium (Mg) and micronutrients: iron (Fe), copper (Cu), manganese (Mn), zinc (Zn) in potato leaf and root tissues were analyzed through Inductively Coupled Plasma Optical Emission Spectroscopy (ICP-OES) as previously described.^21^ The same potato tissues used for qPCR analyses were oven-dried and 0.5 g of dried tissue was ashed at 510 °C for 9 h, allowed to cool, and digested in 10 ml of 1 N HNO_3_ for 1 h. The filtered supernatant was brought to volume (25 ml) and the intensities of atomic emissions at 396.847 nm for Ca, 766.491 nm for K, 279.553 nm for Mg, 238.204 nm for Fe, 327.395 nm for Cu, 257.610 nm for Mn, and 213.857 nm for Zn was measured on an ICP-OES System (Varian Vista Pro CCD Simultaneous ICP-OES attached to Varian SPS 5 Sampler Preparation System, Agilent, USA). Samples were diluted 1:100 in 1 N HNO_3_ prior to Ca, K, and Mg analyses. All containers used for ICP Spectroscopy analysis were acid-washed by soaking overnight in 1 N HNO_3_ before use.

### Statistical analyses

For RNA-Seq analyses, the DESeq R package was used to perform pairwise comparisons. The Benjamini-Hochberg method was used for adjusting *P*-value to control the false discovery rate. *C*_t_ values from qPCR analyses were log transformed and analyzed by Student’s *t*-test at 95% confidence interval (*P*<0.05). The nutrient concentration values were subjected to analysis of variance (ANOVA). Both analyses were carried out using SigmaPlot software Version 11 (Systat Software, San Jose, CA, USA) and means were separated using the Fischer’s Least Significant Difference (FLSD) test at >95% confidence interval.

## Results and discussion

Understanding the global molecular mechanisms involved in plant response to pathogen infection is important in facilitating the efficient development of stable disease resistant or tolerant crops.^22^ In the present study, transcriptional analyses of global gene expression patterns in addition to an evaluation of the nutritional status of healthy or ZC-affected leaf and root tissues were performed to develop comprehensive host response profiles and to help delineate the consensus and central molecular processes associated with potato response to Lso.

### Transcriptional profiling of leaf and root tissues in response to Lso infection

RNA-Seq analyses of leaf and root tissues of healthy and ZC-diseased potato plants yielded 13 606 125 read counts from healthy leaf tissues; 13 297 760 read counts from healthy root tissues; 15 907, 617 read counts from infected leaf tissues; and 16 772, 849 read counts from infected root tissues that matched to the potato genome sequence available in www.ncbi.nlm.nih.gov. Pairwise comparisons identified 133 gene transcripts (120 upregulated; 13 downregulated) in leaf tissues and 243 gene transcripts (35 upregulated; 208 downregulated) in root tissues that were differentially expressed in response to Lso infection.

**Figure 1:**
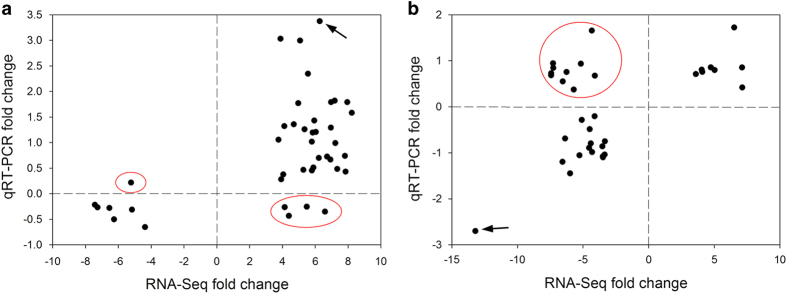
Transcriptional analyses identifying differentially expressed genes in potato tissues in response to Lso infection. (**a**) Putative Lso-mediated differentially expressed gene transcripts in leaf tissues and their expression patterns as detected by RNA-Seq and qPCR analyses. The arrow points to a cysteine proteinase inhibitor (gi|1575307) that was strongly upregulated by both qPCR and RNA-Seq analyses (**b**) Putative Lso-mediated differentially expressed gene transcripts in root tissues and their expression patterns as detected by RNA-Seq and qPCR analyses. The arrow points to a proteinase inhibitor II (gi|21553) that was strongly downregulated by both qPCR and RNA-Seq analyses. The fold changes in ZC-diseased tissues compared to healthy tissues are presented in Log_10_ for qPCR analyses and in Log_2_ for RNA-Seq analyses. The genes enclosed in red circles showed inconsistent expression patterns between RNA-Seq and qPCR analyses.

Based on RNA-Seq results, qPCR primers for 136 differentially expressed genes in leaf and/or root tissues were designed and detected against cDNA samples from all plants used in this study (Supplementary Table S1). Results showed that 36 (88%) out of the 41 genes tested in leaf tissues had consistent expression patterns with results obtained from RNA-Seq analyses, which included a cysteine proteinase inhibitor (gi|1575307) that was shown to be strongly upregulated by both qPCR and RNA-Seq analyses (Figure 1a). Of the 34 genes tested in root tissues, 24 (71%) had a consistent pattern of expression with results obtained from RNA-Seq analyses, which included a proteinase inhibitor II gene (gi|21553) that was shown to be strongly downregulated by both qPCR and RNA-Seq analyses (Figure 1b). These results suggest a major difference in the regulation of gene expression between leaf and root tissues in response to Lso infection. The genes in leaf and root tissues that were consistent in their expression patterns during qPCR and RNA-Seq analyses were considered to be validated and will be henceforth referred to as the differentially produced gene transcripts in response to Lso infection.

**Figure 2:**
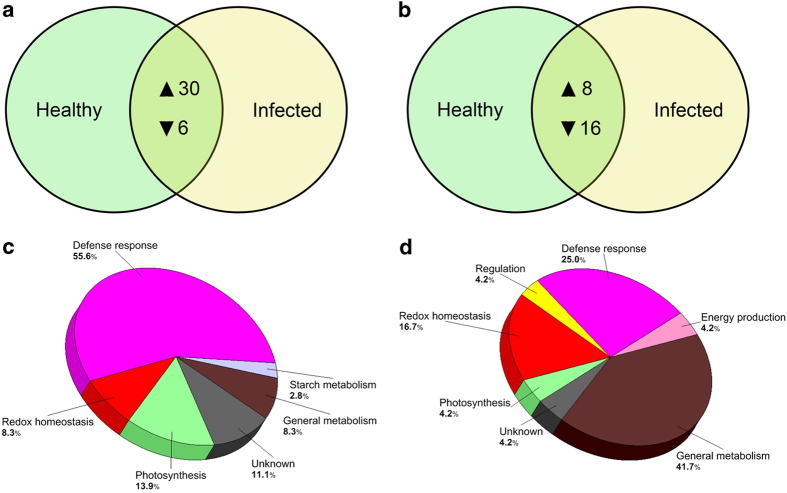
Descriptive/functional classification of differentially expressed gene transcripts in potato tissues in response to putative Lso infection. Venn diagrams show the number of transcripts with increased (▲) or decreased (▼) abundance in leaf (**a**) or root (**b**) tissues from ZC-diseased plants compared to healthy plants. Pie charts show the functional category distribution of differentially produced transcripts in leaf (**c**) or root (**d**) tissues from ZC-diseased plants compared to healthy plants.

Among the differentially expressed gene transcripts identified in leaf tissues, 30 (83%) were upregulated while 6 (17%) were downregulated in response to Lso infection (Figure 2a). Additionally, among the differentially expressed gene transcripts in root tissues, 8 (33%) were upregulated while 16 (67%) were downregulated in response to Lso infection (Figure 2b). Categorization based on functional groups showed that 55.6 and 25% of differentially produced transcripts in leaf and root tissues, respectively, matched to genes associated with defense response (Figures 2c and d).

**Figure 3:**
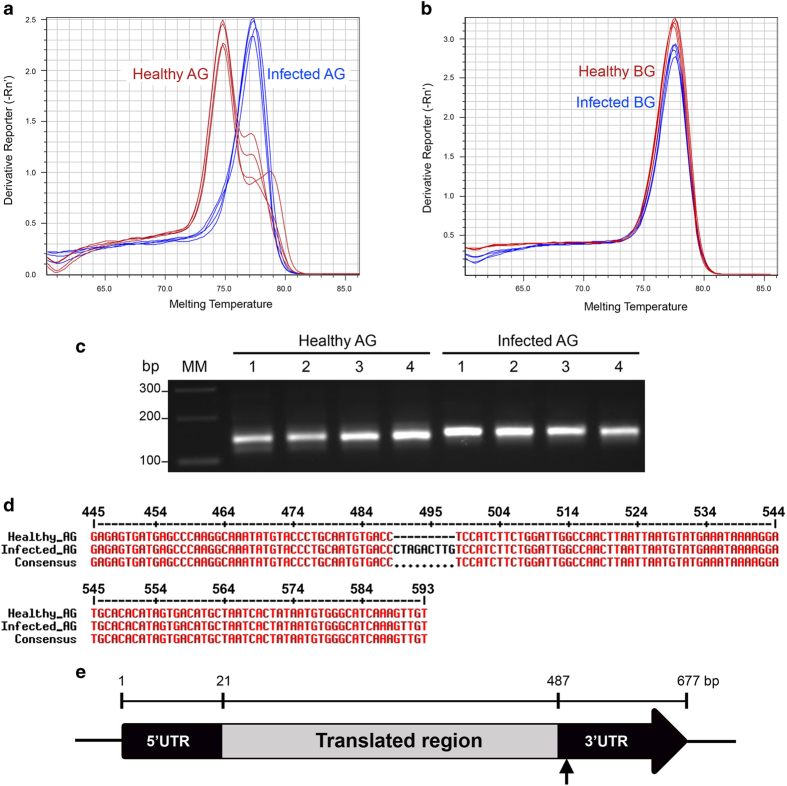
Alternative splicing of a proteinase inhibitor II precursor (PIN2) gene in potato tissues in response to putative Lso infection. (**a**) The melting temperatures of qPCR amplicons of PIN2 in healthy (red lines) and ZC-diseased (blue lines) leaf tissues. (**b**) The melting temperatures of qPCR amplicons of PIN2 in healthy (red lines) and ZC-diseased (blue lines) root tissues. (**c**) Electrophoresis analysis of the qPCR products of PIN2 in leaf tissues from four replicate healthy and infected potato plants. (**d**) Sequencing analyses of the qPCR amplified segment of PIN2 from leaf tissues showing a missing 9 bp region in healthy tissues compared to ZC-diseased tissues. (**e**) Directional alignment of the 677-bp PIN2 gene with arrow pointing to the locus of the missing 9 nucleotides.

Based on qPCR dissociation curves, the amplicons for a proteinase inhibitor II (PIN2) precursor gene (gi|73920934) from ZC-affected leaf tissues had a dissociation temperature of 77.3 °C, which was significantly higher than the 74.8 °C recorded for healthy tissues (Figure 3a). Interestingly, this difference in melting temperatures for PIN2 was not observed in root tissues and the 77.3 °C melting temperature of PIN2 in ZC-diseased leaf tissues was identical to that of both healthy and ZC-diseased root tissues (Figure 3b). Evaluation of PIN2 qPCR amplicons from leaf tissues on an agarose gel showed a slight increase in the size of amplicons from ZC-diseased replicate plants compared to healthy plants (Figure 3c). Sequence analyses and alignment of the amplicons indicated that the sequences from healthy and ZC-diseased leaf tissues at nucleotide positions 445–593 were identical except for 9 nucleotides (5ʹ-CTAGACTTG-3ʹ) that were missing at positions 489–497 in the sequence from healthy leaf tissues (Figure 3d).

Sequence alignment of the 677 bp gene transcript showed that the deletion of 9 nucleotides constitute part of the 3ʹ untranslated region (UTR; Figure 3e). Though not shown, it is important to mention that the sequence of qPCR amplicons for PIN2 in ZC-diseased leaf tissues was identical to that of healthy and ZC-diseased BG tissues.

The production of transcript variants of the same gene in plants, especially when under stress, has been attributed to alternative splicing, a mechanism involved in regulating gene expression.^23^ *Citrus clementina* plants were shown to produce an alternatively spliced variant of defense-related acidic chitinase in response to spider mite (*Tetranychus urticae*) infestation.^24^ Furthermore, though relatively understudied in plants, in animals, the 3′ UTR is frequently implicated as the site of variations due to alternative splicing, which is likely due to its unique involvement in regulatory processes that include cleavage, polyadenylation and alternative polyadenylation.^25,26^ PIN2 is a serine proteinase inhibitor with trypsin and chymotrypsin inhibitory activities and occurs in many Solanaceae plants.^27^ To the best of our knowledge, this study presents the first report showing the involvement of alternative splicing in Lso-mediated gene regulation in potato plants.

### Defense/stress-related proteinase inhibitors, proteases and redox homeostasis-related genes are contrastingly regulated in leaf and root tissues in response to Lso infection

**Figure 4:**
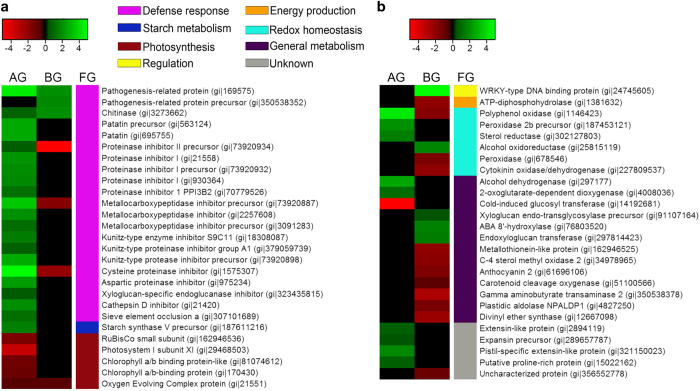
Comparative differentially expressed transcripts in ZC-diseased leaf (AG) and root (BG) tissues. FG denotes the functional group of the transcript (**a**) Defense response-, starch metabolism and photosynthesis-related transcripts that were differentially produced in ZC-diseased AG and BG tissues compared to healthy tissues. (**b**) Regulation-, energy production-, redox homeostasis- and general metabolism-related transcripts that were differentially produced in ZC-diseased AG and BG tissues compared to healthy tissues.

By grouping genes based on sequence homology and functional similarity, genes associated with defense responses, particularly pathogenesis-related (PR) proteins, proteinase inhibitors, chitinase^28^ and patatin^29^ were the most consistently transcriptionally upregulated in leaf and/or root tissues due to Lso infection (Figure 4a). This was expected since PR proteins and chitinases are generally considered to be defense response-related genes ^28–30^ and patatin has been previously shown to be upregulated in potato in response to putative Lso infection.^10^ Additionally, the upregulation of transcripts of a sieve element occlusion gene in leaf tissues (Figure 4a), which could play a role in inhibiting Lso proliferation in phloem tissues, suggest that the innate hypersensitive response mechanism is activated in potato plants in response to Lso infection (Figures 4a and b), albeit insufficient or inefficient in stopping Lso pathogenesis.^31^

Interestingly, proteinase (or protease) inhibitors, which are also associated with defense response, were strongly upregulated in leaf tissues but drastically downregulated in root tissues (Figure 4a). Plant proteinase inhibitors are known to inhibit microbial and pest proteases.^32^ Xu *et al.*^33^ showed that the heterogeneous expression of a phloem-specific *Solanum americanum* proteinase inhibitor II gene inhibited endogenous protease activity in transgenic lettuce (*Lactuva sativa*) plants. Lso is a phloem-limited pathogen, thus the upregulation of PIN2 in leaf tissues may be directly targeted towards Lso proteases or towards endogenous proteases to limit the availability of recycled amino acids to Lso.^32^

However, the downregulation of proteinase inhibitors in root tissues is intriguing and might suggest a source of susceptibility of potato plants to Lso infection. The inhibition of proteinase inhibitor transcripts by *Leptinotarsa decemlineata* regurgitant was observed in *Solanum lycopersicum* ^33^ and the downregulation of proteinase inhibitors was detected in citrus plants affected by citrus sudden death disease.^34^ Such observations are suggested as avenues used by biotic agents to attenuate host defense systems and bolster their ability to invade their hosts.^35,36^ Thus, the differential regulation of proteinase inhibitors (and proteases) in leaf and root tissues (Figure 4a) in response to Lso infection, which we propose to be facilitated by alternative splicing (Figure 3), highlights a potential key molecular process in ZC pathophysiology in potato plants.

Furthermore, while majority of the redox homeostasis-related transcripts (including polyphenol oxidase) identified in leaf tissues were upregulated, in root tissues, except for alcohol dehydrogenase, transcripts for polyphenol oxidase, peroxidase, cytokinin oxidase, and cytokinin dehydrogenase were downregulated (Figure 4b).

Redox homeostasis-related genes, especially superoxide dismutase and peroxidase, typically play antioxidant roles in plants by dissipating the build-up of reactive oxygen species (ROS), which are toxic byproducts of metabolic processes and known to be markedly increased under stress conditions.^37,38^ Thus, unlike other stress response-related genes it is unclear why the general transcriptional responses of redox homeostasis-related genes were inconsistent and disparate between leaf and root tissues. Nonetheless, we suggest that these results help to elucidate the different mechanisms employed by leaf and root tissues in response to putative Lso infection as well as shed light on possible susceptible molecular systems involved in Lso-induced disruption of host defense processes.

Several reports have suggested that polyphenol oxidase, a ubiquitous enzyme in plants that converts phenolics into brown-colored melanins and benzoquinones, plays a role in Lso-induced browning of underground tubers, which is a typical symptom for ZC.^10,11,39,40^ Thus, although transcripts of polyphenol oxidase were upregulated in leaf tissues, the observed downregulation of polyphenol oxidase transcripts in root tissues suggests that further studies might be required to determine the molecular mechanisms responsible for Lso-induced browning of potato tubers.

### A net increase in gene expression but downregulation of photosynthesis processes indicates low resource-use-efficiency in potato tissues infected by Lso

When plants are under biotic stress, photosynthesis is typically inhibited and regulation of gene expression is channeled towards the production of stress-response-related factors at the expense of housekeeping genes/proteins.^41^ This agrees with results in the present study that revealed a concomitant putative Lso-mediated downregulation of photosynthesis-related transcripts, including ribulose-1,5-bisphosphate carboxylase oxygenase (RuBisCO), RuBisCO activase, oxygen evolving complex proteins and chlorophyll a/b-binding protein in leaf tissues (Figure 4a) in potato tissues. Although predominantly underground, the expression of photosynthesis-related transcripts in root tissues is not uncommon as a study by Valkov *et al.*^42^ demonstrated that photosynthesis-related transcripts are expressed in tubers but in significantly lower amounts compared to leaf tissues.

Additionally, when photosynthesis is inhibited, protein production is also reduced since the reducing energy generated in the light-dependent reactions of photosynthesis is important in the *de novo* synthesis of biomolecules, including proteins and carbohydrates.^43,44^ Alvarado *et al.*^10^ reported increase in total protein production in leaves of ZC-affected potatoes. In this study, we showed majority (83%) of the differentially expressed transcripts were upregulated (Figure 2a). Thence, our results suggest a general net increase in gene expression that is associated with putative Lso infection in leaf and root tissues despite the down regulation of photosynthesis-related genes. It is hypothesized that the earlier discussed putative Lso-mediated upregulation of proteinase inhibitors and corresponding downregulation of proteases in leaf tissues might play a role in this phenomenon.

Among the broadly upregulated genes in ZC-diseased tissues compared to healthy tissues included those associated with regulation, particularly the WRKY-type DNA binding protein (Figure 4b). The WRKY transcription factor family is widely involved in regulating plant development and defense response against biotic and abiotic stresses.^45^ In potato plants, a WRKY-like transcription factor was induced by *Erwinia carotovora* and *Phytophthora infestans* infections and was reported to be co-regulated with Class I endochitinase.^46^ Other Lso-mediated upregulated genes identified in this study included genes associated with general metabolism. For example, alcohol dehydrogenase, xyloglucan endo-transglycosylase precursor and endoxyloglucan transferase (Figure 4b) in addition to several transcripts with unknown functions including a putative proline-rich protein, extension-like protein and expansin-like protein (Figure 4b).

Furthermore, an earlier study by Alvarado *et al.*^10^ showed an increase in starch accumulation in leaf tissues in response to Lso infection and suggested that physiological process during the ZC development involves the reprogramming of leaf tissues to mimic root tissues. A hypothesis that fits well with the observation that the 3′-UTR transcriptional variant of PIN2 in ZC-diseased leaf tissues, while different from that of healthy leaf tissues, was identical to those of healthy and ZC-diseased root tissues (Figure 3a). However, the underlying mechanisms responsible for Lso-induced accumulation of starch in leaf tissues are unknown. In the present study, starch metabolism-associated starch synthase (Figure 4a) was upregulated in leaf tissues but not in root tissues, which provides new information on molecular mechanisms associated with Lso-mediated starch anabolism in leaf tissues.^10^ This result is also congruent with recent studies that showed an upregulation of starch synthase in citrus grapefruit and lemon plants in response to Las, the causal agent of citrus HLB,^17,18^ hence may constitute a consensus host molecular response pattern during plant–Liberibacter interactions.

In summary, while putative Lso-mediated downregulation of photosynthesis-related genes was typical, the net increase in gene expression was uncanny and suggests that ZC pathophysiology involves Lso-associated low resource-use-efficiency in potato tissues. To the best of our knowledge this is the first report highlighting a plant–pathogen interaction that involves a net increase in gene expression or in protein production in the case of citrus HLB under limiting photosynthesis.

### Mineral nutrients, particularly K, are actively accumulated in ZC-diseased potato tissues

**Figure 5:**
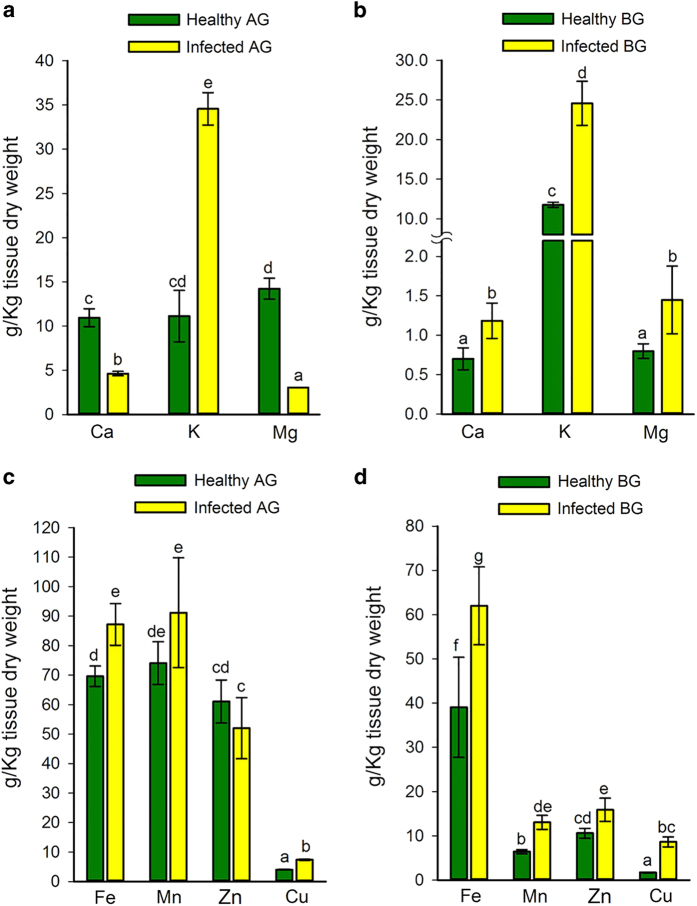
The concentrations of macro- and micronutrients in healthy or ZC-diseased potato plants. Macronutrients calcium (Ca), potassium (K) and magnesium (Mg) concentrations in healthy or ZC-diseased leaf tissues (**a**) or root tissues (**b**). Micronutrients iron (Fe), manganese (Mn), zinc (Zn), and copper (Cu) concentrations in healthy or ZC-diseased leaf tissues (**c**) or root tissues (**d**). Bars with the same lower case letter are not significantly different from each other (*P*>0.05).

Pathogen infection has been reported to affect the nutritional status of plants by reducing or increasing the accumulation of specific nutrients.^15^ In this study, the observed general upregulation of gene transcripts in potato tissues in response to putative Lso infection (described above) suggests that there could be an associated effect on nutrient concentration.^47^ This prompted an investigation into Lso-mediated ramification on the nutritional status of leaf and root tissues of potato plants. Results showed 58 and 79% reductions in the Ca and Mg concentrations, respectively, of ZC-diseased leaf tissues compared to uninfected tissues (Figure 5a). In contrast, there were 69 and 82% increases in the Ca and Mg concentrations, respectively, of ZC-diseased root tissues (Figure 5b) and a similar pattern of putative Lso-induced nutrient accumulation was observed for micronutrients Fe, Mn, Zn, and Cu in leaf and root tissues (Figures 5c and d). The most striking putative Lso-mediated increase in tissue-nutrient concentration was for K, which had 210 and 108% increases in leaf and root tissues, respectively, compared to uninfected tissues (Figure 5a). During periods of stress, plants generally experience growth inhibition and there are reports that in certain situations, growth-inhibited stressed tissues can accumulate more nutrients per unit mass than unstressed tissues.^48,49^ However, in potato plants, increase in nutrient concentrations appears to be unique to putative Lso infection, since symptomatically-similar heat necrosis-affected tubers^50^ showed a concomitant decrease in their nutrient concentrations compared to healthy tubers (Supplementary Figure S1).

Plant nutrients, especially K, are actively involved in gene transcription and translation ^51^ and several metabolically-active proteins (enzymes) depend on the availability of specific nutrients for activation.^52,53^ Similar to ZC-diseased potato leaf tissues, Las-infected citrus leaves showed a distinctive increase in starch synthase expression accompanied by an increase in leaf K concentration,^17,18^ which, based on our studies, are the most consistent co-regulated changes in gene expression and nutritional status between citrus and potato plants in response to their respective putative Liberibacter (Las and Lso) pathogens. Starch synthase requires K for activation,^54^ and it is therefore perfective to suggest that abnormal starch accumulation in leaf tissues of citrus^13^ and potato^10^ tissues associated with Liberibacter infections is facilitated by an increase in K accumulation and a co-regulated increase in the expression of starch synthase.

## Conclusion

In summary, Lso is a phloem-limited bacterial plant pathogen.^6^ Similar to HLB, sieve tubes of ZC-affected potatoes are blocked.^30^ ZC pathophysiology involves an occlusion of phloem vessels by putative Lso that disrupts the source-to-sink flow of photosynthates that conceivably benefits the bacterium but also initiates a systemic signal of starvation in the host, abnormal starch accumulation in above ground leaf tissues and a consequential increase in potassium concentration in tissue. Further investigations to determine the role of alternative splicing in the regulation of proteinase inhibitors in leaf tissues and the potential role of redox homeostasis-related genes in potato susceptibility to putative Lso as well as application of fertilization schemes for nutritional management of ZC are currently being explored.
