# Supplementary figures and images for: Characterization of physiological and molecular processes associated with potato response to Zebra chip disease
Source: Hortic Res. 2017 Dec 6;4:17069–. doi: 10.1038/hortres.2017.69 (PMC5717366; doi:10.1038/hortres.2017.69)

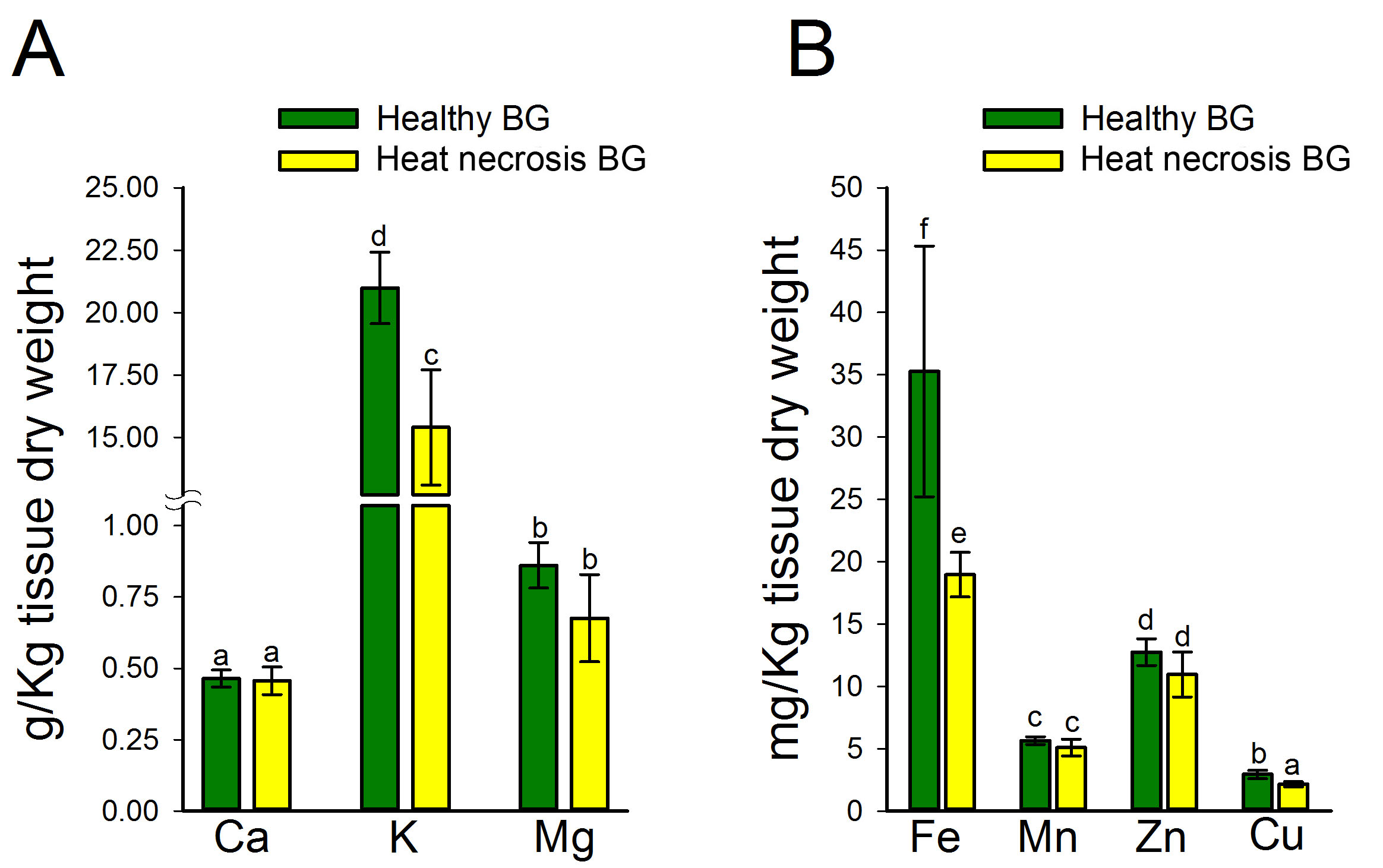

Supplement: Supplementary Figure S1 [file hortres201769-s1.jpg]
