# Supplementary material for: Characterization of physiological and molecular processes associated with potato response to Zebra chip disease
Source: Hortic Res. 2017 Dec 6;4:17069–. doi: 10.1038/hortres.2017.69 (PMC5717366; doi:10.1038/hortres.2017.69)
Supplement: Supplementary Figure Legend [file hortres201769-s2.docx]

**Figure S1.** Nutrient concentrations in below-ground (BG) tissues of healthy or heat necrosis-affected potato plants. (**A**) Macronutrients calcium (Ca), potassium (K) and magnesium (Mg) concentrations in healthy or heat necrosis-affected BG tissues. (**B**) Micronutrients iron (Fe), manganese (Mn), zinc (Zn), and copper (Cu) concentrations in healthy or heat necrosis-affected BG tissues. Bars with the same lower case letter are not significantly different from each other (*P* > 0.05).
