# Supplementary material for: Characterization of physiological and molecular processes associated with potato response to Zebra chip disease
Source: Hortic Res. 2017 Dec 6;4:17069–. doi: 10.1038/hortres.2017.69 (PMC5717366; doi:10.1038/hortres.2017.69)
Supplement: Supplementary Table S1 [file hortres201769-s3.doc]

**Table S1**. List of qRT-PCR primers used in this study and their corresponding genes

| Primer set # | Gene name | Accession # | Forward primer | Reverse primer |
| --- | --- | --- | --- | --- |
| 1 | Kunitz-type protease inhibitor precursor | gi|73920938 | TGTAACGGAATACGCCATCTG | GCATCTCCTCTACCTAAGTCG |
| 2 | Proteinase inhibitor I precursor | gi|73920912 | CATATCCTGCTGACTTTCCTAC | CTTGCTTTCTTGCTTCTTGC |
| 3 | Aspartic proteinase inhibitor homologue | gi|975234 | GGCGTATTCCGTTACAATTCC | TCTCCAACAACATCGTCCATAG |
| 4 | Cysteine proteinase inhibitor 5 precursor | gi|1575313 | CAAGATGAGCGGGACAATACAG | TAAGTCGGAGTCGGATGATGG |
| 5 | Cysteine protease inhibitor | gi|3452386 | CAACTGATGATGCTGGAAAGAAG | TTGATAATGCCTCCGAGTATTGG |
| 6 | Proteinase inhibitor II precursor | gi|73920934 | AGAGAGTGATGAGCCCAAGG | ACAACTTTGATGCCCACATTATAG |
| 7 | Proteinase inhibitor 1 PPI3B2 | gi|70779526 | GCAACTTCCTTTGAAACTCTC | CTCCTTCTCAATTATTTCCTTAGC |
| 8 | Kunitz-type protease inhibitor precursor | gi|73920898 | GAACCGTGTGATGGGAGTATTAC | GGATGGATGTCAGCGAATGC |
| 9 | Putative proteinase inhibitor type II | gi|149785836 | ATTCTCATCCGTCAAGTTAGCC | CAAAGTGCCTGTAGTTTAATGGTG |
| 10 | Proteinase inhibitor II | gi|576528 | CCTGGCTCATCATTCTTTCTTTC | CCTATTCCAAATGTTCTCCGTTTC |
| 11 | Kunitz-type proteinase inhibitor group A1 | gi|379059739 | CAGATGGCGTATTCCGTTAC | GCATTTAGATTTCCGACTTTCC |
| 12 | Kunitz-type protease inhibitor precursor | gi|73920942 | GGCGTATTCCGTTACAATTCC | TCTCCAACAACATCGTCCTTAG |
| 13 | Kunitz-type enzyme inhibitor S9C11 | gi|18308087 | CGGCGATCCAGTCAAGATTG | AGAATGAACTTTACGGGTGTCC |
| 14 | Kunitz-type protease inhibitor precursor | gi|73920898 | CAGTGAATCTCCTCTACCTAAGC | CATCGGAATTGTAACGGAATACG |
| 15 | Proteinase inhibitor I precursor | gi|73920932 | AAAGAAAGTGATGAACCAGAAGTC | AACCAACCACAGGTATGTCTAC |
| 16 | Cathepsin D inhibitor | gi|21420 | AAGGACGATGTTGTTGGAGAC | AAAGAATAGGGCGAGTAATAGGG |
| 17 | Cathepsin D inhibitor | gi|24745610 | TGACCTACCCACTGATACTACTC | CATCGGAATTGTAACGGAATACG |
| 18 | Cysteine proteinase inhibitor 8 precursor | gi|1575307 | GTCATTACACGCACCACATC | CATTATTAACAATCCTCTCATCGG |
| 19 | Cysteine proteinase inhibitor 8 precursor | gi|1575307 | CGTGTTCCGTTCCGTAAGTC | CCTACCTGACCACCAGTTACC |
| 20 | Metallocarboxypeptidase inhibitor IIa precursor | gi|3091283 | CAAGTGTTGGTTCTTCTTTTCG | CAAGCATAGTTGATTTATTATTAGCC |
| 21 | Wound-inducible proteinase inhibitor I | gi|930364 | GCTTGCTAAGAAATAATTGAAAGG | CATGTGGCTGCTTAATTACTTC |
| 22 | Proteinase inhibitor II | gi|21553 | TGTCCTTCGCAAATGAAAATCC | GAATGTGGTAATCTTGGCTATGG |
| 23 | Proteinase inhibitor I | gi|21558 | AACCAACCACAGGTATGTCTAC | AGAACTTCTAAAGGAATTTCAATGC |
| 24 | Proteinase inhibitor I precursor | gi|73920932 | TTGCTAAGGGGATAATTGAGAAGG | CCAACCACAGGTATGTCTACAAC |
| 25 | Metallocarboxypeptidase inhibitor precursor | gi|73920887 | TTGCTATCTTCTTCGTTGTTCTC | AATCGGCGTTTGTGTTGC |
| 26 | Cysteine proteinase inhibitor 8 precursor | gi|1575307 | CAAGATGGCAATCCGCTGAG | CCGACTTACGAACGAACACC |

**Table S1**. List of qRT-PCR primers used in this study and their corresponding genes (*Continued*)

| Primer set # | Gene name | Accession # | Forward primer | Reverse primer |
| --- | --- | --- | --- | --- |
| 27 | Proteinase inhibitor I precursor | gi|73920932 | GGAGTCAAAGTTTGCTCACATC | CACCAAGATTCAGATTCCGATTC |
| 28 | Proteinase inhibitor II | gi|21555 | TCTATATGTTGATGCTTTGGGTTG | TTTGGGTCAGATTCTCCTTCG |
| 29 | Metallocarboxypeptidase inhibitor | gi|2257608 | TTGCTATCTTCTTCGTTGTTCTC | CAATCGGCGTTTGTGGTG |
| 30 | Xyloglucan-specific endoglucanase inhibitor protein 2 | gi|323435815 | TGATGATGTATTGGTGATTGGTTC | CTAAAGCAAGTTGATTTGGAATGG |
| 31 | Threonine deaminase | gi|21582 | GATGTGTATGATGAAGGAAGGAAC | CTCTGCTAACTCTGTAACTTTGTG |
| 32 | Patatin precursor | gi|563124 | GAATTGGATGCTAAGATGTATGAC | CAACGCTAATGGATAATAATGCC |
| 33 | Patatin | gi|695755 | TGTTGATGGTGCTGTTGCTAC | AGTGCCAGTGCCTAATGAGAG |
| 34 | Sieve element occlusion a | gi|307101689 | AAGCAAGCAATAGAAGAAAGCAG | ATTACACAATTTGGGAAGAAGACG |
| 35 | 42KDa chitin-binding protein | gi|269935956 | CAGGGACGATGCGGATGG | GGTGGAGGAGGAGGTGAGG |
| 36 | Leucine aminopeptidase | gi|21486 | CTTCCAGTAACTCTCCTCCATAG | GGTGCTATTGTGATTGCTCTTG |
| 37 | Class I chitinase | gi|3273662 | TGTGGTAGGCAGGCAGGAG | GGTAGGTGGTTTGGGAGAAGG |
| 38 | Putative non-specific lipid transfer protein | gi|21952513 | TGGCTCCTTGTCTCCCTTATC | ACCGTGGAACAGTCAGTGG |
| 39 | Pistil-specific extensin-like protein | gi|321150023 | AACCACCGTCTCCTCTTCC | GCTTCACTACCGCTCCTTG |
| 40 | Sterol reductase | gi|302127803 | AATGCCGCCAACTTGAGC | CTTCCTTCTCTGTCTTCCTTCC |
| 41 | Putative proline-rich protein | gi|15022162 | CACCACCAGCACAACCAAC | GCATCCAAGTCTACAAGTCCTC |
| 42 | Allene oxide synthase | gi|56605357 | CACTTTCCCTCTACCTTACTTCC | CCTGATGTTCCGATCCACTTG |
| 43 | Extensin-like protein | gi|2894119 | CAAGTGGTCGTGGCAAGAG | ATCGTGGTGGTGGAGGTG |
| 44 | UDP-galactose:solanidine galactosyltransferase | gi|82802846 | CGTGAACTCCGTCCTGATTGC | GATTAGGCTGCTTGTGAGGTCTG |
| 45 | Alcohol dehydrogenase | gi|297177 | AATGGAAGTTCGTCTCAAG | TAAGAGGCTACACATATTGC |
| 46 | Peroxidase 2b precursor | gi|187453121 | ATGCTGTTGTCTTGAGTGG | AGTGGCTGATACCGATGG |
| 47 | Polyphenol oxidase | gi|1146425 | CTATACCATTAGCAGCATCGG | AGCAATATACTCCTCATCAACAG |
| 48 | Peroxidase precursor | gi|350539340 | TTGGAGTTGCCTTGGTTGGAG | CCTTGTTGGTGAATTGTGGTCTC |
| 49 | Endo-beta-1,4-D-glucanase precursor | gi|350535029 | GTGGACCTGATGCCTATGAC | TGATTGTAACCGCTGTGACC |
| 50 | Expansin precursor | gi|289657787 | GCATTAAGCACAGCATTATTCAAC | CACCAACCACCATTATCATTAGG |
| 51 | Extensin | gi|296400 | CCAGTGAAGCCATACCATCC | GGAGACGGAGACTTGTAAACG |
| 52 | Starch synthase V precursor | gi|187611216 | CGGATTATCTCGTGCCTTGC | CTCCAATGCCACAGACAACC |

**Table S1**. List of qRT-PCR primers used in this study and their corresponding genes (*Continued*)

| Primer set # | Gene name | Accession # | Forward primer | Reverse primer |
| --- | --- | --- | --- | --- |
| 53 | Cold-induced glucosyl transferase | gi|14192681 | CACAAGGTCATATCAATCCATCTC | GAATGCCGCCAAGTTTAAGC |
| 54 | Haloacid dehalogenase | gi|242381584 | CGACTGGCTGTTCTGACTC | ATTAGGCTTCTCTGCTTCTACC |
| 55 | Xyloglucan endo-transglycosylase precursor | gi|91107164 | AGAACTTCCACACCTACTCAATC | CCCAATCATCAGCATTCCAAAG |
| 56 | Chitinase | gi|21494 | ACCAGCAGGGAGAGCAATAG | GCAGAATCAGCAGCAGAGG |
| 57 | WRKY-type DNA binding protein | gi|24745605 | TAAGAGGCTACACATATTGC | AAGTTATCGGTAGATTTGTCAATGG |
| 58 | Beta-fructofuranosidase | gi|397630 | CTAAACACCCACTCCACTCAG | TCAAGTCTCAATCCTTTCCATCC |
| 59 | Alcohol NADP+ oxidoreductase | gi|25815119 | CTCGTCAGATTCCTACTTACAATG | CCTCCAAGACCAACAACACC |
| 60 | Pathogenesis related protein PR-1 | gi|6066749 | AGTGGCTGATACCGATGG | CGCACACTTGTCCGCTTG |
| 61 | ABA 8'-hydroxylase CYP707A2 | gi|76803520 | ATCACAACCCAGAGTTCTTTCC | ACTACCAGATCCTACCACTTCC |
| 62 | Pathogenesis-related protein P2 precursor | gi|350538352 | ATGCGGTAGATGCTTGAGG | CCAATCCATTAGTGTCCAATCG |
| 63 | Endoxyloglucan transferase | gi|297814423 | TGTTGGCAGAGAAGAAAGATATGG | TGGCTTAGATGGGAAGTCACC |
| 64 | Peroxidase | gi|678546 | CCTGTCTTAACTTCAACTCTTCC | AGGTCTATTCCAATCCGATGC |
| 65 | Metallothionein-like protein | gi|162946525 | AAAGCAGACGGAGGAAATGG | ATACATAGAAGAAAGGCACATACC |
| 66 | Dehydration-responsive element-binding protein | gi|358008881 | AGAGGAGGAGGAGGAGGAG | CCGCTATCACCGACTAACAG |
| 67 | Cysteine protease inhibitor | gi|3452386 | CAACTGATGATGCTGGAAAGAAG | TTGATAATGCCTCCGAGTATTGG |
| 68 | Hemoglobin | gi|27085252 | CGAAGACTATTGTTATTGTTGTTG | AGTATGGTGTGGCTGATGAG |
| 69 | Metallocarboxypeptidase inhibitor IIa precursor | gi|3091283 | AAATTGGATCTGCGTGTTGC | CATTCTCCTTGTGGTTATTGCTG |
| 70 | Glucose acyltransferase | gi|4101704 | GTATGACCCGCACCCTTAAC | TGAGCACTGAAGAATGGATAGAC |
| 71 | Cytokinin riboside 5'-monophosphate phosphoribohydrolase LOG6 | gi|384081613 | GGAAGTAGTCAAGGCAAGAAGAG | TGTGAAACCAAACCCATAAGGC |
| 72 | Putative Rieske Fe-S protein precursor | gi|37222948 | AGATGATAGAGTGCCTGATATGG | TCCCTTTAGTCCTTGTGTGAG |
| 73 | 23 kDa oxygen evolving protein of photosystem II | gi|1771777 | ATTGGCTCTTACTCTCCTCATTG | GCTTGAATCCATCTCCATTGTATG |
| 74 | Ferredoxin--NADP(+) reductase | gi|2225992 | GAACCTCCATCATCTCCACTG | TTATTCACAATCACACCTTCATCC |
| 75 | C-4 sterol methyl oxidase 2 | gi|34978965 | AGCATCTTCAACTTTCTGTAACC | CGAGGCACATTCTGGATACC |
| 76 | X intrinsic protein | gi|309385604 | GTTCAACCTTGCTCTTCTACAC | GACGCTCGCCATACATCC |
| 77 | Proteinase inhibitor II | gi|21525 | TAATAGCGGTTGTTCTGTTTGTG | GCAATAAGGAAGATGGTAATGAGG |
| 78 | Anthocyanin 2 | gi|61696106 | CCTCGTCCTAATCCTCATCTAC | CATTGAACTCCATCGTCTATCG |
| 79 | Polygalacturonase-1 non-catalytic subunit beta precursor | gi|350538028 | ATTCGGTCTCGGATTCAACTC | ACTTTGGACAGGCGTATTGC |

**Table S1**. List of qRT-PCR primers used in this study and their corresponding genes (*Continued*)

| Primer set # | Gene name | Accession # | Forward primer | Reverse primer |
| --- | --- | --- | --- | --- |
| 80 | Serine carboxypeptidase-like 42-like isoform 1 | gi|356552778 | AAGATTCTGTTGTGCCATTGC | GTTGCCATACTCTGTTTGCC |
| 81 | Aspartic proteinase inhibitor | gi|21408 | CAGTGAATCTCCTCTACCTAACG | CATCGGAATTGTAACGGAATACG |
| 82 | Carotenoid cleavage oxygenase | gi|51100566 | TGCTTCCTTCCAGTGTAGTTC | ATATTCCACAACGCCAATGC |
| 83 | Chlorophyll a-binding protein | gi|170393 | ACTACCATATCTCCGCCTCTG | CACTGGATGTAGAAAGAAGAAACC |
| 84 | Cytokinin oxidase/dehydrogenase | gi|227809537 | AGCCGTTCTTGGTGGATTAG | ATTCTTGGTCTTGTGTAAATGTTG |
| 85 | 33kDa precursor protein of oxygen-evolving complex | gi|21551 | CATTCACCGTCAAGGCAGAG | AGCAGCGTAATCAATTCCATCC |
| 86 | Chloroplast pigment-binding protein CP29 | gi|110377765 | ACTCCGTTTCAGCCTTACAG | CCAATCACAAGAACCTCAATCC |
| 87 | Omega-3 fatty acid desaturase | gi|350537636 | GTGATGTGGTTGGATTCTGTC | AAGGTGATAATGAGGTATTTGAGG |
| 88 | Light dependent NADH:protochlorophyllide oxidoreductase 1 | gi|9049761 | TTACAGGAAACACTAACACTTTGG | CTCTTCGTGGTATCGTCTATGG |
| 89 | Gamma aminobutyrate transaminase 2 | gi|350538378 | GCTATCAACAAACTCGGTAGAAAG | TCCAAGGTCATAAGTGCTCAAAG |
| 90 | Ferredoxin I | gi|14041723 | GCTGGTTCTTGCTCATCTTG | AATGGTAACATCACACTTTGGG |
| 91 | Gibberellin 2-oxidase 1 | gi|152003422 | GCAGGCAATGAGTAACGGAAG | GGGACAACCTATTATCACCAAGC |
| 92 | NADPH:protochlorophyllide oxidoreductase | gi|21068892 | CGAGATTGTTGCTTGATGACTTG | TTAAACCTCCCGCCATACCC |
| 93 | Ribulose-1,5-bisphosphate carboxylase/oxygenase small subunit | gi|162946536 | CGTGTGCCCTTCCTCTTTAC | TCCTCCTCGTCTCCTCTCC |
| 94 | Chloroplast chlorophyll a-b binding protein | gi|48375047 | GTTCAAAGCAGGAGCACAAATC | TGGTAGGGTCATCAGCAAGG |
| 95 | Chlorophyll a,b binding protein type I | gi|511152 | GAAGGTTACAGAGTTGGTGGAG | CAGCAATGTGGTCGGAAAGG |
| 96 | Plastidic aldolase NPALDP1 | gi|4827250 | CGCCTTCCATCCGTCTCC | TGCCTCAGTGTTCTCCATTCC |
| 97 | Dihydroflavonol 4-reductase | gi|33772291 | TCATTAGCATCATACCACCACTG | GCACCATCTTAGCCACATCG |
| 98 | Photosystem I subunit XI | gi|29468503 | TCCAACTTGCCTGCCTACC | CTGCTGCTGCCAATGAACC |
| 99 | Divinyl ether synthase | gi|12667098 | AAGTCCGCTGTGTCCATATTG | CACCACCCTCTTCTTTAATAACG |
| 100 | Non-phosphorylating glyceraldehyde dehydrogenase | gi|1842114 | CCATCACCAACACGACCTTC | TTCACTGGCGGAGACACC |
| 101 | Flavonoid 3'-hydroxylase | gi|5921646 | GTTGTAAAGTCAGCCCAAATGC | AGCCCAAAGTTGATGTGAAAGG |
| 102 | Chalcone synthase 1a | gi|1292923 | CCGTGGAGGAGTATCGTAAGG | GCTCAGTCTTGTGCTCACTATTAG |
| 103 | Mg protoporphyrin IX chelatase | gi|2318136 | CTGTGATGCTCCTGGTGTTG | AAGGTTGGTTGGATCTGAATCG |
| 104 | Chalcone isomerase | gi|299889034 | GGCTATTGTTAATGCTCCAGTTG | AGTGCTTCTTCCTCATCTTCTTC |
| 105 | Flavanone 3 beta-hydroxylase | gi|21392364 | AGAGCCTGACCTTACCCTTG | CATTCTTGAACCTCCCATTGC |
| 106 | Flavonol synthase | gi|1039356 | GGTCCAAGCAATATCGTCAATAAC | TCTCAATCACTTCATCAGGTATCC |

**Table S1**. List of qRT-PCR primers used in this study and their corresponding genes (*Continued*)

| Primer set # | Gene name | Accession # | Forward primer | Reverse primer |
| --- | --- | --- | --- | --- |
| 107 | Chlorophyll a-b binding protein 3C-like | gi|81074612 | ACATTCGCTAAGAACCGTGAG | ATCCAGCCTTGAACCATACAG |
| 108 | Flavonoid 3',5'-hydroxylase | gi|56269730 | CTATGGACCTCGTTGGAAGTTG | TCAGCAATCACCACACATTCG |
| 109 | Chlorophyll a/b-binding protein | gi|170389 | GCACCAAGCATAGCCCAAC | GAGCCAAACACCCATCATACC |
| 110 | Chlorophyll a/b-binding protein | gi|170430 | ATTAAGAACGGTAGACTTGCTATG | TGCTTCTGCTTCTCACTTGG |
| 111 | Glyceraldehyde-3-phosphate dehydrogenase | gi|170236 | GAGGAAGGAAGAACAAGAGGATAG | TGGCAATGACATCAAGAGGTG |
| 112 | Glutathione S-transferase | gi|304366637 | TACTCCAACTCCTCGTATTTCC | GCAAAGCCACCTTCATTCATC |
| 113 | Flavonoid 3-glucosyl transferase | gi|62112650 | ATCAACAAAGACCTCAAATCAAAG | TCCAACGCTTCTGCTATCG |
| 114 | Chalcone synthase 2 | gi|1470059 | GCGAAGGTGCTATTGATGGTC | AATGCCTAACGGTTGGAATGC |
| 115 | Proteinase inhibitor II | gi|21553 | TTGGGTCAGATTGTCCTTCG | TCTTGCTTACTATACTGTTCTTGG |
| 116 | 2-oxoglutarate-dependent dioxygenase | gi|4008036 | GGCGGACCTTCTTTCAAACTG | ACACTTCGTTCTTCACCATTAGC |
| 117 | Polyphenol oxidase | gi|404586 | TTCCTTCACCACCAACAACTC | GCACCATAAAGACCTCCTAACC |
| 118 | Rhamnose:beta-solanine/beta-chaconine rhamnosyltransferase | gi|82590366 | ATGTATGGAAGTGGCTGGTTG | CCGTCTGAGGAAGTTGGATTAC |
| 119 | Polyphenol oxidase | gi|1146423 | CGCCTAAGCCTGATGATATGG | TCTATAAGCACCGTTACAATAAGC |
| 120 | Patatin precursor | gi|73426682 | ATGATGCTTCTGAGGCTAATATGG | ATTTACAACTACAACCCGAGACC |
| 121 | Pathogenesis-related protein | gi|169575 | GATTGAAGGAGATGTTCTTGGAG | GATTGGCGAGGAGGTATGC |
| 122 | ATP-diphosphohydrolase | gi|1381632 | ATGGTGGTGGTGGTGATGG | TTGTTTGGCAAGCAACTTTAGC |
| 123 | Proteinase inhibitor II | gi|21555 | GAATGTGGTAATCTTGGCTATGG | CGCAAATGAATGTCCCGTTAG |
| 124 | Metallocarboxypeptidase inhibitor | gi|2257606 | GCTATCTTCTTCGTTGTTCTCTTG | ACCACTGGAGGACCTTCTTC |
| 125 | Glutamine synthetase GS1 | gi|209529863 | GGACCACAGGGACCATACTAC | CACGACAACTCCAGCAATCTC |
| 126 | Actin 1 | gi|21533 | TGGGATGATATGGAGAAGATTTGG | GAGACAAGACTGCCTGAATAGC |
| 127 | Actin 2 | gi|21535 | GAAGTAACATAAGATGGCAGAAGG | CAAGAGTCCGAGCAACATAGG |
| 128 | Actin 5 | gi|21543 | TGGCAGACGGAGAGGATATTC | GGCAACAAGGTAATAAGCATCAG |
| 129 | Actin 7 | gi|1498371 | CTGGTATTGCTGATAGGATGAG | TGCTGGAATGTGCTGAGG |
| 130 | Actin 8 | gi|1498379 | CAGGTGTGATGGTTGGTATGG | CTTAGGATTGAGAGGTGCTTCG |
| 131 | Β-tubulin | gi|609267 | ATGTTCAGGCGCAAGGCTT | TCTGCAACCGGGTCATTCAT |
| 132 | Ef1α | gi|24745944 | ATTGGAAACGGATATGCTCCA | TCCTTACCTGAACGCCTGTCA |
| 133 | L2 | gi|39816659 | GGCGAAATGGGTCGTGTTAT | CATTTCTCTCGCCGAAATCG |

**Table S1**. List of qRT-PCR primers used in this study and their corresponding genes (*Continued*)

| Primer set # | Gene name | Accession # | Forward primer | Reverse primer |
| --- | --- | --- | --- | --- |
| 134 | 18S rRNA | gi|511154 | GGGCATTCGTATTTCATAGTCAGAG | CGGTTCTTGATTAATGAAAACATCCT |
| 135 | aprt | CK270447 | GAACCGGAGCAGGTGAAGAA | GAAGCAATCCCAGCGATACG |
| 136 | Cyclophilin | gi|4559301 | CTCTTCGCCGATACCACTCC | TCACACGGTGGAAGGTTGAG |
